# Silencing of Mitochondrial Trifunctional Protein A Subunit (HADHA) Increases Lipid Stores, and Reduces Oviposition and Flight Capacity in the Vector Insect *Rhodnius prolixus*

**DOI:** 10.3389/finsc.2022.885172

**Published:** 2022-06-09

**Authors:** Daniela S. Arêdes, Iron F. De Paula, Samara Santos-Araujo, Katia C. Gondim

**Affiliations:** Instituto de Bioquímica Médica Leopoldo de Meis, Universidade Federal do Rio de Janeiro, Rio de Janeiro, Brazil

**Keywords:** mitochondrial trifunctional protein, β-oxidation, oogenesis, flight, fat body, *Rhodnius prolixus*

## Abstract

*Rhodnius prolixus* is an obligatory hematophagous insect, vector of Chagas disease. After blood meal, lipids are absorbed, metabolized, synthesized, and accumulated in the fat body. When necessary, stored lipids are mobilized, transported to other organs, or are oxidized to provide energy. Mitochondrial β-oxidation is a cyclic conserved pathway, where degradation of long-chain fatty acids occurs to contribute to cellular energetic demands. Three of its reactions are catalyzed by the mitochondrial trifunctional protein (MTP), which is composed by hydroxyacyl-CoA dehydrogenase trifunctional multienzyme complex subunits alpha and beta (HADHA and HADHB, respectively). Here, we investigated the role of HADHA in lipid metabolism and reproduction of *Rhodnius prolixus* females. The expression of HADHA gene (*RhoprHadha*) was determined in the organs of starving adult insects. The flight muscle and ovary had higher expression levels when compared to the anterior and posterior midguts or the fat body. *RhoprHadha* gene expression was upregulated by blood meal in the flight muscle and fat body. We generated insects with RNAi-mediated knockdown of *RhoprHadha* to address the physiological role of this gene. *RhoprHadha* deficiency resulted in higher triacylglycerol content and larger lipid droplets in the fat body during starvation. After feeding, lifespan of the knockdown females was not affected, but they exhibited a decrease in oviposition, although hatching was the same in both groups. Silenced females showed lower forced flight capacity than the control ones, and their fat bodies had lower gene expression levels of Brummer lipase (*RhoprBmm*) and long-chain acyl-CoA synthetase 2 (*RhoprAcsl2*). Taken together, these findings indicate that HADHA is important to guarantee successful reproduction and efficient mobilization of lipid stores during starvation and flight.

## Introduction

Lipids are an important source of energy, and degradation of fatty acids is essential for the maintenance of energy homeostasis. Mammals mobilize lipid depots in moments when there is an increased demand or limitation of substrates, as fasting or exercise, but not only at these moments, and lipid degradation constitutively occurs in various cell types (1). Mitochondrial β-oxidation is the major pathway for fatty acids degradation in different organisms. Inside mitochondria, acyl-CoAs (acyl-coenzyme A esters, activated fatty acids) are degraded by a recurring sequence of four enzymatic reactions which shortens the fatty acid by releasing two carbon atoms as acetyl-CoA at each round (2, 3). The first step is the acyl-CoA dehydrogenation by an acyl-CoA dehydrogenase. The next three steps - a hydration followed by a second dehydrogenation and a thiolytic cleavage to yield acetyl-CoA - are catalyzed by the mitochondrial trifunctional protein (MTP), attached to the mitochondrial inner membrane. This enzyme is a hetero-octamer composed of four α and four β subunits, encoded by the *Hadha* (hydroxyacyl-CoA dehydrogenase trifunctional multienzyme complex subunit alpha) and *Hadhb* (hydroxyacyl-CoA dehydrogenase trifunctional multienzyme complex subunit beta) genes, respectively (4, 5). Each α-subunit contains the long-chain enoyl-CoA hydratase (LCEH) and long-chain 3-hydroxyacyl-CoA dehydrogenase (LCHAD) domains. In the same way, the long-chain 3-ketoacyl-CoA thiolase domain (LKAT) is located in both β-subunits.

MTP has been widely studied in mammals. More than twenty defects related to one of the subunits of the enzyme in different organs have been described, as cardiomyopathy, liver disease, rhabdomyolysis and sudden death in humans (6, 7). MTP α-subunit knockdown caused hepatic steatosis, hyperinsulinemia and an increase in the activity of antioxidant enzymes in adult mouse model (8), in addition to hypoglycemia, neonatal death and liver necrosis (9). Defects in the thiolase activity, present in the β subunit of the protein, led to the development of hypoparathyroidism and childhood polyneuropathy (10).

In insects and other invertebrates, knowledge about the β-oxidation pathway in general is extremely scarce. In the worm *Caenorhabditis elegans*, loss-of-function mutations of *kat-1* (ketoacyl thiolase) gene, an orthologue of the mammalian 3-KAT, resulted in premature aging and shorter lifespan indicating a connection between aging and deficient fat oxidation (11). In *Drosophila melanogaster* flies, mutants for MTP α or β-subunits had shorter lifespan, decreased locomotor activity, and laid fewer eggs. They also had higher contents of triacylglycerol (TAG), acylcarnitines and hydroxyacylcarnitines, suggesting that in both cases fatty acid β-oxidation was compromised (12). Although mutation of the MTP α-subunit seemed to produce stronger phenotypes, these results indicated that dysregulation of one of the subunits may somehow affect the other one, as it was reported for humans (13).

*Rhodnius prolixus* is an obligatory hematophagous hemipteran, vector of Chagas disease which affects 6-7 million people worldwide (14). This insect feeds on blood during the entire life cycle, nymphs and adults. After a blood meal, as digestion slowly occurs, the adult female accumulates lipids in the fat body and produces the eggs. Lipids from the ingested blood are metabolized in the midgut, secreted to the hemolymph and are transported to the other organs, as the fat body and the vitellogenic ovaries, by a major circulating lipoprotein, lipophorin (15–19). The fat body incorporates lipids from the hemolymph, but also synthesizes fatty acids *de novo*, using amino acids as substrates (20), and stores TAG in lipid droplets, in a process that is regulated by insulin signaling (21). After about two weeks, when digestion is close to completion, TAG amounts in the fat body start to decrease (17). Thus, in periods of energy demand, lipids are mobilized from lipid droplets by neutral lipolysis, as described for mammals and other insect species (22, 23), and/or by lipophagy, as we have shown to occur in *R. prolixus* (24). These events lead to the release of free fatty acids which, after activation by acyl-CoA synthetases, are potential substrates to mitochondrial β-oxidation (25, 26). All the genes related to β-oxidation are present in *R. prolixus* genome, with the only exception of long-chain acyl-CoA dehydrogenase (LCAD) gene, which was not found in any other analyzed insect genome (27). Orthologue genes that encode the MTP α and β subunits were identified: *RhoprHadha* and *RhoprHadhb* (27). However, there is no information about the relevance of HADHA and HADHB activities for metabolic homeostasis in *R. prolixus* or any hematophagous insect. Thus, in this study we have investigated the importance of HADHA function in *R. prolixus*, in order to better understand mitochondrial β-oxidation pathway in insects, and we showed that, when *RhoprHadha* expression was inhibited, TAG mobilization, reproduction and flight capacity were affected.

## Material and Methods

### Insects

The insects were from a colony of *Rhodnius prolixus* maintained at 28 ± 2°C and 75 ± 10% relative humidity, and 12 h/12 h light and dark cycles. Adult insects were fed on the outer ears of live rabbits in three-week intervals. Animal care and experimental protocols were approved by Committee for Evaluation of Animal Use for Research from the Federal University of Rio de Janeiro (CAUAP-UFRJ), process number 01200.001568/2013-87, order number 149/19, in accordance with the NIH Guide for the Care and Use of Laboratory Animals (ISBN 978-0-309- 18663-6). Experimental insects were adult mated females, fed in three-week intervals, in starvation condition (18 – 21 days after a blood meal) or after the second or third feeding cycle as adults.

### Extraction of RNA and cDNA Synthesis

At appropriate days after blood meal, adult females were dissected, the organs (anterior and posterior midguts, abdominal fat body and ovary) were collected and washed. To obtain ovarian follicles at different stages of growth, ovaries were dissected and follicles were removed in 0.15M NaCl under a stereomicroscope, grouped according to their length into four categories: 0.5, 1.0, 1.5 and 2.0 mm. Recently laid eggs were collected 0 – 24h after oviposition. Organs were immediately homogenized (pools of three organs, or two eggs) in TRIzol Reagent (Invitrogen, Carlsbad, CA, USA) for total RNA extraction. After quantification with a NanoDrop Lite Spectrophotometer (Thermo Fisher Scientific, Waltham, MA, USA), the integrity and quality of the RNA samples were analyzed by electrophoresis on 2% agarose gel (UBS, Cleveland, OH, USA), and RNA was considered intact when the 18S rRNA band was observed. The RNA samples were treated with DNase I in a final volume of 10 μl (Thermo Fisher Scientific, Waltham, USA), to remove contaminants from genomic DNA, and were used for cDNA synthesis with the High Capacity cDNA Reverse Transcription Kit (Applied Biosystems Inc., Foster City, USA).

### Quantitative PCR (qPCR)

The qPCR were done in a StepOnePlus thermocycler (Thermo Fisher Scientific), using GoTaq qPCR Master Mix (Promega, Madison, WI, USA). The reaction mixture contained three pmol of sense and antisense primers (**Supplementary Table 1**) in a final volume of 15 μl, as follows: 10 min at 95°C, followed by 40 cycles of 15 s at 95°C and 1 min at 60°C, and a dissociation curve. *Rhopr18S* gene (GenBank ID: AJ421962) amplification was used for normalization, as previously described (28), and its amplification was constant under our experimental conditions. For the blanks, the cDNA was replaced by nuclease-free water, and the Cq (quantification cycle) values obtained for the blanks were at least ten units above the experimental points. The ΔΔCq values were calculated from the obtained Cq values as described elsewhere (29), submitted to Grubb’s test for outliers detection (30), and were used for statistical analyses. The relative expression values (2^-ΔΔCq^) were used only for data plotting.

### RNA Interference

The double strand RNAs for the *RhoprHadha* gene (VectorBase Gene ID: RPRC006225; 27) was produced with the MEGAScript™ T7 High Yield Transcription Kit (Thermo Scientific), using the primers listed in **Supplementary Table 1**. The unrelated bacterial MalE gene (GenBank ID: 948538) was used as a control dsRNA (31). One µg of each dsRNA (dsHadha or dsMal) was injected into the insect hemocoel, on the eighteenth day after a blood meal (starvation condition), using a 10 µl syringe (Hamilton Company, Reno, USA). Insects were either kept in unfed condition and were analyzed three days after dsRNAinjection (21 days after blood meal, starvation condition), or were fed three days after dsRNA injection, and analyzed at different days after blood meal. Inhibition of *RhoprHadha* gene expression was confirmed by qPCR as described above at each experiment.

### Determination of TAG and Protein Contents

Adult females were injected with 1 µg of dsRNA on the eighteenth day after feeding (starvation condition). Three days later, the abdominal fat body, flight muscle and ovary were dissected from control and knockdown insects. Alternatively, the females were fed three days after dsRNA injection, and the abdominal fat body was dissected on the seventh day after blood meal. Eggs laid by the fed females were collected (0–24h after oviposition). The samples were washed in PBS (10 mM phosphate buffered saline, pH 7.4, 0.15 M NaCl) and individually homogenized in 200 µl of PBS (for eggs, pools of two in 100 µl) in a Potter-Elvehjem tube, for TAG and protein quantification. TAG content was determined with the Triglicérides 120 colorimetric kit (Doles Reagentes, Goiânia, Brazil), which quantifies the glycerol released from TAG after hydrolysis by a lipase. Total protein content was determined (32) using bovine serum albumin as standard.

### Protein Digestion Measurement

Three days after dsRNA injection, females were fed and dissected at days 1, 2, 4, 7 and 10 after feeding, to follow the protein digestion. The midguts (anterior plus posterior midguts, including luminal contents) were collected and individually homogenized in a Potter-Elvehjem tube in 500 µl of PBS for total protein content determination as described above.

### Oviposition, Hatching, and Lifespan

Adult females were injected with dsRNA, and three days later were fed and separated in individual vials. The laid eggs were daily collected and counted until the end of the laying cycle. Hatching was also accompanied, and nymph morphology was visually inspected. Time from oviposition until nymph eclosion was registered. For comparison of the adult lifespan, these females were daily observed until all the insects had died.

### Nile Red Staining of the Lipid Droplet

Adult females were injected with dsRNA on the eighteenth day after a blood meal (starvation condition), and three days later the abdominal fat bodies were dissected and stained with Nile Red and DAPI (Sigma-Aldrich, Saint Louis), as previously described (33). The fat bodies were incubated for 15 min in 1 mg/ml Nile Red and 2 mg/ml DAPI made up in 75% glycerol. Tissues were mounted in 100% glycerol and immediately imaged in a Leica TCS-SPE laser scanning confocal microscope (Leica Microsystems, Wetzlar, Germany) where peripheral regions of the fat bodies were analyzed. The excitation wavelengths used were 543 nm for Nile Red and 280 nm for DAPI. The average diameters of the lipid droplets were obtained from four representative images for each insect, five insects per condition, using DAIME image analysis software after edge detection automatic segmentation (34). The lipid droplets diameters were plotted in a frequency histogram (bin width: 1).

### Hemolymph Protein Composition

Adult females were injected with 1 µg of dsRNA on the eighteenth day after feeding (starvation condition), and three days later were fed. Seven days after blood meal, hemolymph was individually collected in the presence of phenylthiourea, centrifuged at 6000*g* for 5 min, and the supernatants were used for protein concentration determination (32). Alternatively, for protein composition analysis, the hemolymph samples (1 µl), were subjected to SDS-PAGE (10%), followed by densitometry of vitellogenin (Vg) bands, with ImageJ software version 1.50i (NIH Image, Bethesda, MD, USA), with background corrections. Egg samples were used to confirm Vg identification.

### Flight Activity

Adult females were injected with dsRNA on the eighteenth day after feeding (starvation condition). Three days later, the insects were subjected to a forced flight assay on an apparatus that forces the insects to fly (35), and was previously used for flight evaluation in *R. prolixus* (24, 36). Briefly, the insect is hung by a thread, attached to the dorsal surface of its thorax, in front of a fan-generated continuous air flow. The flight times until complete exhaustion were recorded for each insect. The insects were considered completely exhausted once they stopped flying for more than 30s despite the continuous stimulation by the air flow.

### Statistical Analyses

Student’s *t*-test was used for comparison between two conditions, and One-way ANOVA followed by Tukey’s test or Two-way ANOVA followed by Sidak’s post-test for more than two conditions. Differences in survival curves were analyzed using the Log-Rank test, and egg hatching rates were compared by the Chi-square (χ^2^) test. In qPCR experiments, the ΔΔCq values, calculated from Cq values, were used for statistical analyses. Results were obtained from at least three independent determinations and differences were considered significant at *p <* 0.05. Statistical analyses were performed using Prism 8.0 software.

## Results

**Figure 1:**
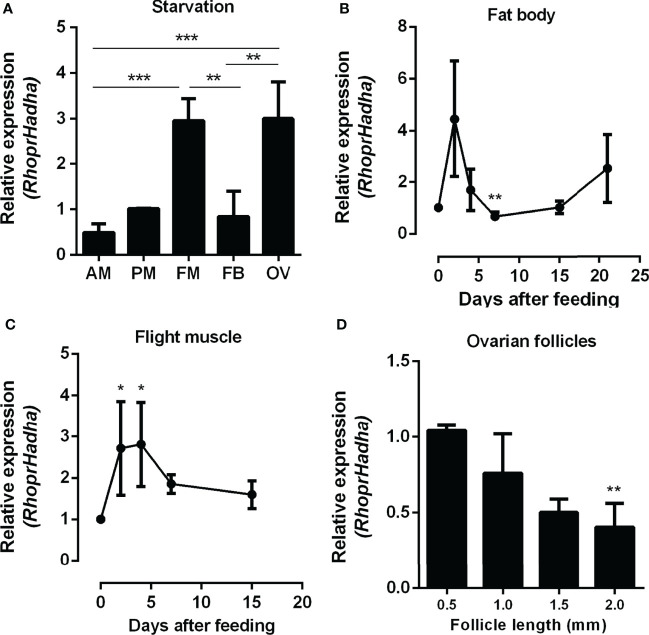
*RhoprHadha* relative gene expression in the insect organs. **(A)** Adult females were dissected before feeding (starvation condition), the organs were isolated and total mRNA was extracted from the samples. Alternatively, adult females were dissected before feeding (day 0), and on different days after a blood meal, and total mRNA was extracted from the fat body **(B)** and flight muscle **(C)**. **(D)** On the fourth day after blood meal the ovaries were dissected, follicles were separated according to their sizes (0.5, 1.0, 1.5 and 2.0 mm length). *RhoprHadha* mRNA levels were determined in samples by qPCR, using the *Rhopr18S* expression as reference gene. Results are means ± SEM and were analyzed by ANOVA followed by Tukey’s post-test. **(A)** (**) and (***): significantly different with *p* < 0.01, and 0.001, respectively (n=3). **(B, C)** (*) and (**): significantly different from days 0 and 2 with *p* < 0.05, and 0.01, respectively (n=4-6). **(D)** (**) significantly different from 0.5 mm with *p* < 0.01 (n=5). AM, anterior midgut; PM, posterior midgut; FM, flight muscle; FB, fat body; OV, ovary.

As a first step to investigate the role of HADHA in *R. prolixus* metabolism, *RhoprHadha* transcript levels were determined in the organs of adult females during starvation, when β-oxidation pathway is expected to have a relevant participation in energy provision. At 21 days after blood meal (starvation condition), the flight muscle and ovary had higher transcript levels, when compared with other organs during this period (**Figure 1A**). The fat body, which is a central organ for lipid metabolism, had lower *RhoprHadha* expression levels in starvation, similar to the anterior and posterior midguts. *RhoprHadha* gene expression was also compared at different days after feeding, as it might be controlled by the nutritional condition. In both the fat body and the flight muscle, *RhoprHadha* mRNA levels showed an increase after blood meal, and then decreased again, confirming that feeding somehow regulates *RhoprHadha* mRNA levels (**Figures 1B, C**). Oocytes were compared according to their developmental stage, and *RhoprHadha* expression was measured in ovarian follicles (containing the oocyte coated by the follicular epithelium) with different lengths: pre-vitellogenic (0.5 mm), vitellogenic (1.0 and 1.5 mm) and chorionated (2.0 mm) (**Figure 1D**). This gene was highly expressed in pre-vitellogenic oocytes, and decreased as the follicle grew, reaching the lower expression levels in mature chorionated oocytes.

**Figure 2:**
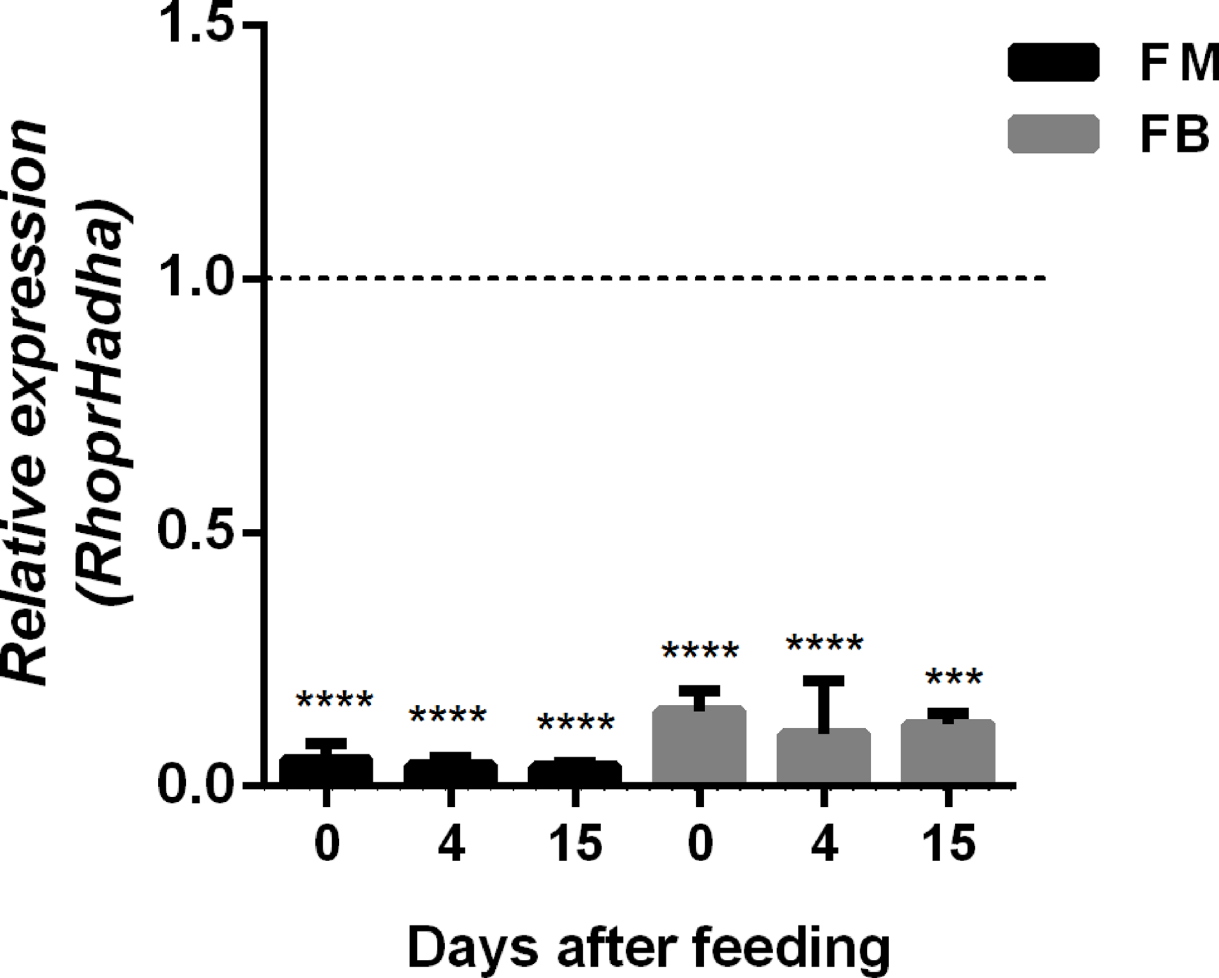
Inhibition of *RhoprHadha* gene expression by RNAi. Fasted adult females (18 days after blood meal) were injected with 1 ug of dsRNA for *RhoprHadha* or *Mal* (control), and were dissected three days later (day 0, starvation condition). Alternatively, the insects were fed three days after dsRNA injection and dissected on days 4 and 15 after feeding. Total RNA was extracted from the flight muscle and fat body, and the *RhoprHadha* mRNA levels were determined by qPCR, using the *Rhopr18S* expression as reference gene. Expression levels are relative to those from control insects (dashed line). The bars are means ± SEM (n=4). (***) and (****): significantly different from dsMal by Student’s *t*-test with *p* < 0.001, and 0.0001, respectively. FM, flight muscle; FB, fat body.

**Figure 3:**
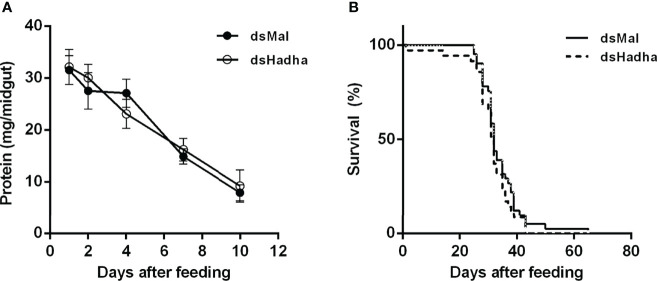
*RhoprHhada* knockdown does not affect digestion or lifespan. Fasted adult females were injected with 1 ug of dsHhada or dsMal (control) and were fed three days later. **(A)** Insects were dissected on different days after feeding, and the total protein amount in the midgut was determined. The symbols are means ± SEM, *p* > 0.05 by Two-Way ANOVA, n = 12. **(B)** After blood meal, insect mortality was daily monitored. *p* > 0.05 by Log-Rank test, n = 45.

In order to try to understand the participation of HADHA in *R. prolixus* biochemistry and physiology, *RhoprHadha* expression was inhibited by dsRNA injection (dsHadha) into adult females. Transcript levels were determined in unfed females (day 0; starving condition) and at days four and 15 after blood meal, to check whether gene silencing lasted during the time when phenotypes were evaluated (**Figure 2**). In both flight muscle and fat body *RhoprHadha* expression was largely inhibited before feeding, and it remained low up to 15 days after blood meal. As it was possible that *RhoprHadha* knockdown affected blood digestion, the midgut luminal total protein content was determined at days after feeding, as a surrogate for the digestive process, and no difference was observed in the dsHadha-treated females (**Figure 3A**). Surprisingly, *RhoprHadha* knockdown did not affect the insect longevity in starvation condition (**Figure 3B**).

**Figure 4:**
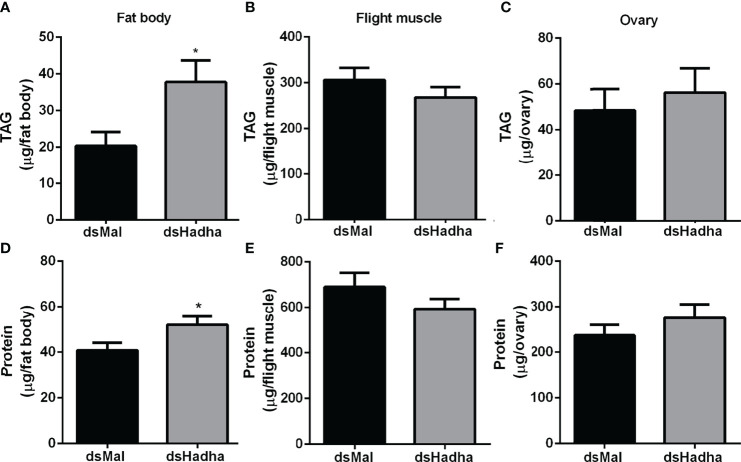
*RhoprHadha* knockdown leads to increased lipid and protein contents in the fat body during starvation. Starving adult females were injected with 1 μg of dsHhada or dsMal (control), and were dissected three days later. The fat bodies **(A, D)**, flight muscles **(B, E)** and ovaries **(C, F)** were individually homogenized and total TAG and protein contents were determined. Graphs show means ± SD (n =12–27). (*): significantly different from dsMal by Student’s *t*-test with *p* < 0.05.

The effect of *RhoprHadha* silencing on the insect lipid stores was evaluated during starvation (21 days after feeding), and TAG content was higher in the fat body of the treated females when compared to the control ones, but no difference was detected in the flight muscle or ovary (**Figures 4A–C**). In the same way, the amount of total protein in these same insects was higher in the fat body of the silenced females, but not in the other organs (**Figures 4D–F**).

**Figure 5:**
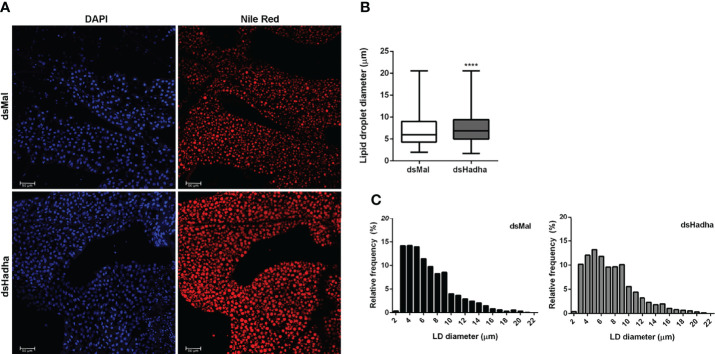
*RhoprHadha* knockdown leads to larger lipid droplets. Adult starving females were injected with 1 μg of dsHhada or dsMal (control) and were dissected three days later. **(A)** The lipid droplets (LDs) were stained with Nile Red, observed under the laser scanning confocal microscope. DAPI-stained nuclei are also observed. Bars: 50 µm. **(B)** Maximum diameters of LDs were determined from five insects per group (four images for each insect), in two independent experiments. Results are means ± S.D. for 6000 LDs per group. (****): significantly different from dsMal by Student’s *t*-test with *p <* 0.0001. **(C)** Diameter distribution histograms of LDs from panel B.

In insects, as in other organisms, TAG is stored in lipid droplets (LDs) which also contain phospholipids, cholesterol and proteins (37, 38). These organelles are present in variable numbers and sizes, according to nutritional status and other conditions, and result from a balance between lipid synthesis and degradation (39). Thus, the LDs from the fat body were analyzed during starvation to verify whether they were also altered in the knockdown females, in accordance with the higher amount of TAG in the fat body of these insects. We found that *RhoprHadha* knockdown females presented larger LDs, despite the large internal variation in LD diameter, as detected by Nile Red staining (**Figures 5A, B**). The control females had a larger proportion of LDs with smaller diameters, between 2 and 7 µm, than the silenced ones (~ 64% versus ~57%, respectively) (**Figures 5C**), indicating that inhibition of *RhoprHadha* expression compromised lipid mobilization from the cell lipid depot.

**Figure 6:**
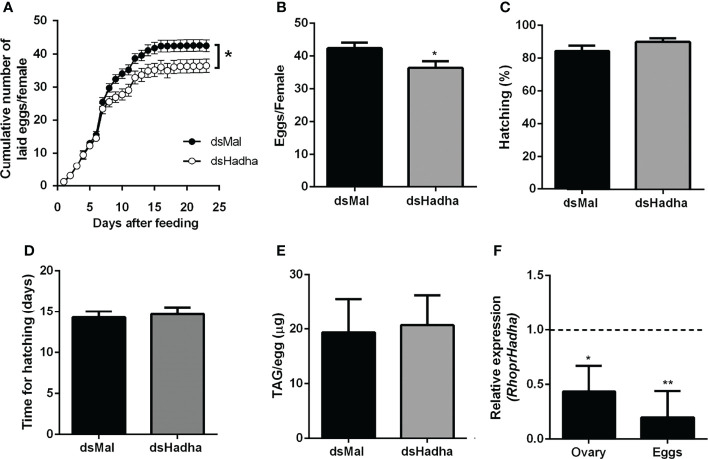
*RhoprHadha* knockdown affects oviposition but not hatching. Adult fasting females were injected with 1 ug of dsHadha or dsMal and were fed on the third day post-injection. **(A)** Cumulative oviposition was daily monitored. Graphs show means ± SD (n = 45 females). (*): *p* < 0.05 by two-way ANOVA. **(B)** The total number of eggs per female was also recorded; *p* < 0.05 by Student’s *t*-test. **(C)** Hatching percentage was determined for laid eggs. **(D)** After eggs were laid, time until nymph hatching was determined. Bars are means ± SD (n = 37- 46 eggs). **(E)** TAG content was determined in recently laid eggs. Bars are means ± SD (n = 7). **(F)** *RhoprHadha* mRNA levels were determined in the ovaries at day 21 after feeding, and in recently laid eggs, using the *Rhopr18S* expression as reference gene. Expression levels are relative to those from control insects (dashed line).The bars are means ± SEM (ovary: n=3; eggs: n = 4). (*) and (**): significantly different from dsMal by Student’s *t*-test with *p* < 0.05, and *p* < 0.01, respectively.

**Figure 7:**
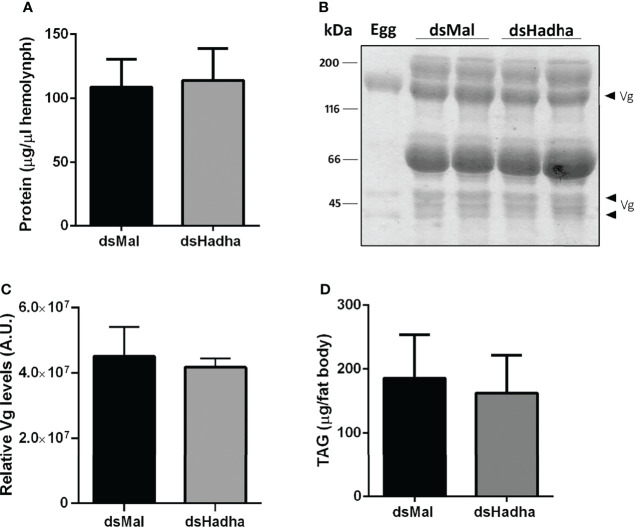
Inhibition of *RhoprHadha* gene expression does not affect lipid accumulation or hemolymph protein composition. Adult fasting females were injected with 1 ug of dsHadha or dsMal and were fed on the third day post-injection. Seven days after blood meal, hemolymph was collected and the fat bodies were dissected. **(A)** Total protein concentration in the hemolymph. Graph shows means ± SD (n = 9). **(B)** Representative SDS-PAGE (10%) analysis of hemolymph (1 μl), followed by densitometry of Vg proteins **(C)**. Bars are means ± SD (n = 7). An egg sample was used to confirm identification of Vg bands. **(D)** Fat bodies were individually homogenized and TAG content was determined. Graph shows means ± SD (n = 14). Vg: vitellogenin.

Although there was no difference in the protein and TAG contents in the ovary of starving females after dsHadha treatment, the number of laid eggs after blood meal was reduced, and knockdown females produced fewer eggs (**Figures 6A, B**). However, once they were laid, they hatched. The nymph eclosion rates were the same in both groups, hatching was not delayed in the silenced eggs, and the egg TAG content was not altered (**Figures 6C–E**). Importantly, *RhoprHadha* expression in the ovary remained inhibited at 21 days after blood meal, after completion of the oviposition cycle, as well as in the eggs laid by the knockdown females (**Figures 6F**). Next, hemolymph was analyzed in order to determine whether *RhoprHadha* knockdown affected vitellogenin production, what could result in fewer laid eggs. No difference was observed in total protein concentration or in the protein composition (or vitellogenin amount) in the hemolymph of silenced females (**Figures 7A–C**). In the same way, the fat bodies of the silenced females had the same TAG content as the control ones at the seventh day after blood meal, confirming that lipid synthesis and accumulation were not affected by *RhoprHadha* inhibition (**Figure 7D**).Thus, the decrease in oviposition observed in the knockdown females was not due to a limitation of substrates for vitellogenesis, such as lipids and yolk proteins.

**Figure 8:**
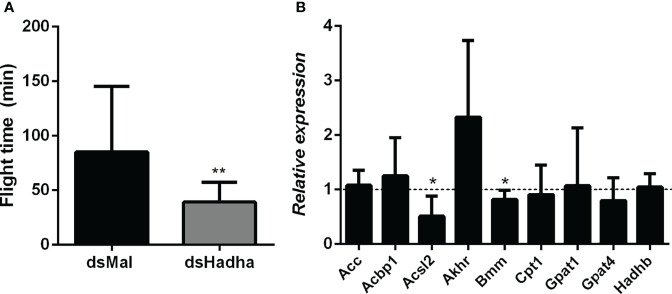
Decreased flight capacity and expression of other lipid-related genes in *RhoprHadha* silenced females. Adult fasting females were injected with 1 ug of dsHadha or dsMal, and three days later (starvation condition) they were subjected to a forced-flight assay. **(A)** The time of flight activity until exhaustion was individually recorded. Graphs show means ± SD (n = 9). (**): *p* < 0.01 by Student’s *t*-test. **(B)** At day 21 after feeding (starvation condition), total RNA was extracted from the fat body of injected females, and the cDNA levels were determined by qPCR, using specific primers designed for target genes using the *Rhopr18S* expression as reference gene. Gene expression levels are relative to each control value (dashed line). Graph shows means ± SEM of 4-8 independent determinations. (*): significantly different from dsMal by Student’s *t*-test with *p* < 0.05. *Acbp1*: acyl-CoA binding protein 1, *Acc*: acetyl-CoA carboxylase; *Acsl2*: long chain acyl-CoA synthetase 2; *Akhr*: adipokinetic hormone receptor; *Bmm*: brummer lipase; *Cpt1*: carnitine palmitoyltransferase 1; *Gpat1* and *Gpat4*: glycerol-3-phosphate acyltransferase 1 and 4, respectively; *Hadhb:* β subunit from mitochondrial trifunctional protein.

As flight is an energy demanding activity, we considered that the inhibition of *RhoprHadha* gene expression might disturb flight capacity. In this way, insects were subjected to a forced flight experiment, and the knockdown females showed a decrease of about 50% in the flight time until exhaustion (**Figure 8A**). The expression levels of some other genes involved in lipid metabolism, which are part of either synthesis or degradation pathways, were measured in the fat body after *RhoprHadha* inhibition. Noteworthy, the genes *RhoprAcsl2* (long chain acyl-CoA sintetase 2) and *RhoprBmm* (brummer lipase) were downregulated after *RhoprHadha* knockdown (**Figure 8B**). In *R. prolixus*, the *RhoprAcsl2* silencing resulted in the inhibition of β-oxidation, showing that ACSL-2 has a central role in the activation of fatty acids for this pathway (25). Bmm is the insect orthologue of ATGL (adipose triglyceride lipase) (27, 40), which has a central role in lipolytic mobilization of stored lipids (41). In this way, upon *RhoprHadha* disruption, genes of other proteins that take part in lipid mobilization and oxidation may also be regulated. Interestingly, the *RhoprHadhb* gene expression was not altered by *RhoprHadha* knockdown, although HADHB and HADHA are both subunits that form the MTP. The expression of the other investigated genes was not affected.

## Discussion

β-oxidation is a fatty acid degradation cyclic pathway, which has as products acetyl-CoA, NADH and FADH_2_ (2, 3). The mitochondrial trifunctional protein (MTP) is a multienzyme complex in this pathway, where alpha and beta subunits catalyze three consecutive reactions. The alpha subunit (HADHA) has the hydratase and dehydrogenase activities, whereas the beta subunit (HADHB) has the thiolase activity. In invertebrates, very few studies have investigated the importance of MTP, and none in a hematophagous insect. In order to investigate the role of this protein in the kissing bug *R. prolixus*, we measured *RhoprHadha* gene expression in the insect organs, and inhibited it with the injection of dsRNA to analyze resulting effects in lipid metabolism and the insect physiology.

The determination of *RhoprHadha* transcript levels in the different organs during starvation revealed some interesting results. First, the ovaries from unfed females had high expression of this gene, in a moment when there is no vitellogenesis taking place, as oocytes will start to develop after blood meal (42). In line with this observation, after feeding, the vitellogenic ovaries showed high *RhoprHadha* transcript contents in the initial, pre-vitellogenic follicles, suggesting the possibility that the ovaries start to produce part of gene products to be used by the growing oocytes before the blood meal.

On the other hand, the fat body, which is a central organ in insect lipid metabolism (26, 37) showed lower transcript levels, similar to the midgut. However, it was responsive to the nutritional condition, and increased after feeding. Accordingly, it was previously shown that the knockdown of *RhoprGpat1*resulted in an increase in mitochondrial fatty acid oxidation in *R. prolixus*, indicating that Gpat1, the first enzyme in the glycerol-3-phosphate pathway for the synthesis of TAG is important to channel fatty acids towards lipid synthesis and away from β-oxidation (43). In this way, the importance of this Gpat1 role is now better understood, as expression of both genes seem to respond to feeding in a similar way (44).

The flight muscles of the triatomines *R. prolixus* and *Triatoma infestans* contain large amounts of lipids, which may be used for flight and general locomotion (45). However, the events of lipid accumulation and utilization by this tissue are still unclear. In some insects, such as *Manduca sexta* and *Locusta migratoria*, during flight, diacylglycerol is released in the hemolymph by the fat body after the hydrolysis of stored TAG, and is transported by lipophorin to the insect muscle fibers, where fatty acids are oxidized for ATP formation, a process that is essential for muscle contraction (26, 46). Here, we found not only that *RhoprHadha* had a high expression level in the flight muscle, but that it was upregulated by the blood meal, suggesting a highly oxidative pattern. Nevertheless, flight muscle TAG content was not affected by *RhoprHadha* silencing.

Surprisingly, inhibition of *RhoprHadha* gene expression did not affect lifespan, differently from *D. melanogaster* mutants for the HADH subunits alpha or beta (genes *Mtpα* or *Mtpβ*), which had reduced lifespan (12). *R. prolixus* is an obligatory hematophagous insect and it is possible that an increase in the oxidation of other substrates, as amino acids, may counterbalance a deficiency in lipid oxidation, but more studies are necessary to clarify this point. In *Aedes aegypti*, proline fuels flight, acting as an efficient respiratory substrate in the flight muscle (47, 48).

The fact that the silenced females had a higher TAG content in the fat body during starvation, when compared to the control ones, indicated that lipid mobilization was impaired, as a consequence of β-oxidation disturbance, and that the fatty acid oxidation machinery somehow connects to and affects the main insect TAG reservoir under fasting conditions. In support of this possibility, the LDs were larger in the dsHadha treated insects, indicating a lower TAG utilization. Similar results were also observed in *R. prolixus* females silenced for either *RhoprAtg6* or *RhoprAtg8*, which caused lipophagy inhibition, confirming the existing crosstalk between the various routes involved in lipid utilization. In *D. melanogaster*, the *Mtpα* mutants also showed reduced TAG mobilization during starvation (12) and, in this aspect, insects responded alike mammals to the HADHA deficiency, as the same lipid accumulation profile was observed in the liver of mice with defects in this MTP subunit (8, 9).

Lipids are very important for reproduction, and they account for about 30 – 40% of an insect egg dry weight (49, 50). They are transported from the midgut, fat body and oenocytes to the ovaries by lipophorin, and are accumulated by growing oocytes, where they are used for oocyte maturation and embryo development (26, 51). In *Culex quinquefasciatus*, egg lipids provide about 90% of the energy to sustain embryonic development (52). Because *RhoprHadha* gene expression was high in the ovary and ovarian follicles, we considered that its knockdown could affect reproduction. Indeed, the silenced females laid fewer eggs than the control ones, reinforcing the central role of fatty acid β-oxidation for insect reproduction, although the effect was not very dramatic. As hemolymph protein composition and lipid synthesis by the fat body were not affected, egg laying was probably decreased due to energetic limitations. In *D. melanogaster*, the knockout of *Mtpα* also led to lower egg laying and, interestingly, although it was a more drastic treatment, where the flies were knockout for this gene, and not knockdown as described here, oviposition was still approximately 60% of control females (12). Inhibition of *RhoprHadha* gene expression did not compromise the embryo development or the eclosion of the nymphs. Although TAG present in *R. prolixus* eggs is consumed during embryogenesis, about half of its initial content is not used and is found associated with the first instar nymphs after eclosion (18). Thus, it is possible that other substrates play significant roles in egg energy supply, and this may help embryos in silenced eggs to develop. It was a possibility that digestion was affected in the knockdown females, what could limit the availability of substrates for ovarian growth, but this was not the case and digestion occurred normally in both insect groups. The efficacy of *RhoprHadha* inhibition obtained in the ovary after dsRNA injection was lower than in the other organs, due to unknown reasons. Also, we have no information about the remaining HADHA protein contents in the various organs after gene knockdown.

The forced flight capacity was reduced in the knockdown females, probably due to a decrease in ATP generation by fatty acid oxidation to sustain flight. On the other hand, TAG content in the flight muscle was not higher in the dsHadha-treated insects, what would indicate lower lipid utilization, but it is possible that flight is mostly fueled by lipids from the fat body, as it was previously suggested for *R. prolixus* (36), and is known to occur in other insects (26, 46). This observed decrease in forced flight capacity, as well as in spontaneous locomotion, was also observed in *R. prolixus* females and nymphs after inhibition of lipophagy (24), reinforcing the importance of lipids for flight muscle contractile activity in this insect.

In humans, mutations in MTP α-subunit (HADHA) reduced expression and increased breakdown of the β-subunit (HADHB) (13), but we did not observe a similar effect in this study. The inhibition of *RhoprHadha* gene expression did not affect *RhoprHadhb* transcript levels. On the other hand, it is noteworthy that the impairment of β-oxidation by *RhoprHadha* knockdown resulted in changes in the expression levels of other lipid-related genes, *RhoprAcsl2* and *RhoprBmm* in the fat body. It is possible that after *RhoprHadha* knockdown, free fatty acids accumulate, which may somehow inhibit *RhoprAcsl2* expression, as Acsl2 is the enzyme that activates fatty acids, released by TAG hydrolysis, and directs them to β-oxidation (25). Also, it is possible that the diminished utilization of fatty acids by that pathway causes the decrease in *RhoprBmm* transcription, as this enzyme catalyzes the first step of lipolysis at the LDs, during TAG mobilization (40). However, more studies are necessary to investigate these hypotheses and to unravel the mechanisms involved in this regulation.

## Data Availability Statement

The datasets presented in this study can be found in online repositories. The names of the repository/repositories and accession number(s) can be found in the article/supplementary material.

## Ethics Statement

The animal study was reviewed and approved by Committee for Evaluation of Animal Use for Research from the Universidade Federal do Rio de Janeiro, CEUA-UFRJ.

## Author Contributions

DA designed and conducted the experiments, analyzed the results, and wrote the manuscript. IP analyzed the results and revised the manuscript. KG designed the experiments and wrote the manuscript. SSA conducted the experiments and revised the manuscript. All authors contributed to the article and approved the submitted version.

## Funding

This work was supported by grants from Conselho Nacional de Desenvolvimento Científico e Tecnológico (CNPq); Fundação de Amparo à Pesquisa do Estado do Rio de Janeiro (FAPERJ); and Coordenação de Aperfeiçoamento de Pessoal de Nível Superior (CAPES; Finance Code 001). KG is a member of the Instituto Nacional de Ciência e Tecnologia em Entomologia Molecular (INCT-EM).

## Conflict of Interest

The authors declare that the research was conducted in the absence of any commercial or financial relationships that could be construed as a potential conflict of interest.

## Publisher’s Note

All claims expressed in this article are solely those of the authors and do not necessarily represent those of their affiliated organizations, or those of the publisher, the editors and the reviewers. Any product that may be evaluated in this article, or claim that may be made by its manufacturer, is not guaranteed or endorsed by the publisher.

## Abbreviations

Acbp1; acyl-CoA binding protein 1; Acc, acetyl-CoA carboxylase; Acsl2, long chain acyl-CoA synthetase 2; Akhr, adipokinetic hormone receptor; ATGL; adipose triglyceride lipase; Bmm, brummer lipase; Cpt1, carnitine palmitoyltransferase 1; CoA, coenzyme A; Gpat1, glycerol-3-phosphate acyltransferase 1; Gpat4, glycerol-3-phosphate acyltransferase 4; HADHA, hydroxyacyl-CoA dehydrogenase trifunctional multienzyme complex subunit alpha; HADHB, hydroxyacyl-CoA dehydrogenase trifunctional multienzyme complex subunit beta; kat, ketoacyl thiolase; LCAD, long-chain acyl-CoA dehydrogenase; LCEH, long-chain enoyl-CoA hydratase; LCHAD, long-chain 3-hydroxyacyl-CoA dehydrogenase; LKAT, ketoacyl-CoA thiolase; LDs, lipid droplets; MTP, mitochondrial trifunctional protein; TAG, triacylglycerol; Vg, vitellogenin.
