# Supplementary material for: Silencing of Mitochondrial Trifunctional Protein A Subunit (HADHA) Increases Lipid Stores, and Reduces Oviposition and Flight Capacity in the Vector Insect Rhodnius prolixus
Source: Front Insect Sci. 2022 Jun 9;2:885172. doi: 10.3389/finsc.2022.885172 (PMC10926480; doi:10.3389/finsc.2022.885172)
Supplement: Supplementary file 1 [file Table_1.pdf]

Supplementary data

Supplementary Table 1: Primer sequences used in the present study.

Primer	Sequence (5' - 3')
**Primers used in qPCR reactions**	
RhoprAcbp_F	GGGGACTGTAATACGAGCAA
RhoprAcbp_R	TTCAATCCATAAGATGCAATCA
RhoprAcc_F	TGGGCTGGAACCGTAGTTGCG
RhoprAcc_R	TGCGGGATCGGCTGGAAGTTGT
RhoprAcsl_F	TAGCCGTAATGGCAGAACGC
RhoprAcsl_R	CCATGGGCAGCTAATTCTGC
RhoprAKHr_F	TTCTATTCGCATGCACCAAC
RhoprAKHr_R	ACTAGTGCGCGAGTTGTTTG
RhoprBmm_F	ACATGGATGGAGGATTCAGTG
RhoprBmm_R	GGGCATATATCGGTTTCACC
RhoprCpt1_F	AAACACCACATGGCCAAACT
RhoprCpt1_R	GAAACGCCGTATCCATCATC
RhoprGpat1_F	TTGTCTGCGACGAACAAGGA
RhoprGpat1_R	AACCGTCGGGTTGCTTCTCT
RhoprGpat4_F	TCTGCCGGTGGTATTCACGA
RhoprGpat4_R	GTCCACTGGTTTGTATTGGAGGA
RhoprHadha_F	TTGGCATGCACTATTTCTCICCAG
RhoprHadha_R	ACTTTACCCGGTTTGAGACCTACA
RhoprHadhb_F	TGAGAGATCCTCCCCAGTTG
RhoprHadhb_R	CATGAAGCTTTTGCAGGACA
Rhopr18S_F	TCGGCCAACAAAAGTACACA
Rhopr18S_R	TGTCGGTGTAACTGGCATGT
**Primers used for dsRNA synthesis**	
RhoprHadha_F	CCGTGAAGTAGCGTCGGAAT
RhoprHadha_R	AGCAATAAAGCACCCAGGCT
T7 minimal	TAATACGACTCACTATAGGG

F, forward
R, reverse
